# Compositional profile of barley landlines grown in different regions of Gilgit‐Baltistan

**DOI:** 10.1002/fsn3.2215

**Published:** 2021-03-24

**Authors:** Abid Hussain, Sartaj Ali, Azhar Hussain, Zubair Hussain, Muhammad Faisal Manzoor, Abid Hussain, Amjad Ali, Talat Mahmood, Kashif Sarfraz Abbasi, Emad Karrar, Maqsood Hussain, Tajudin

**Affiliations:** ^1^ Department of Agriculture and Food Science Karakorum International University Gilgit Pakistan; ^2^ School of Food and Biological Engineering Jiangsu University Zhenjiang China; ^3^ School of Food Science and Engineering South China University of Technology Guangzhou China; ^4^ Nuclear Institute of Food and Agriculture Tarnab Peshwar Pakistan; ^5^ Institute of Food & Nutritional Sciences PMAS‐Arid Agriculture University Rawalpindi Pakistan; ^6^ Department of Food Engineering and Technology Faculty of Engineering and Technology University of Gezira Wad Medani Sudan; ^7^ Mountain Agriculture Research Center (MARC) Juglote, Gilgit Pakistan

**Keywords:** barley landlines, functional properties, Gilgit‐Baltistan, nutritional composition

## Abstract

The current investigation was performed to explore the nutritional and functional composition of four landlines of barley denoted as LB_1_ (Gilgit), LB_2_ (Nagar), LB_3_ (Skardu), and LB_4_ (Shigar) from different regions of Gilgit‐Baltistan. The samples were examined for nutritional profile and antioxidant attributes. Total phenolic values and total flavonoid results were in the range of 1.2 to 3.1 mg/g and 0.41 to 0.55 mg/g, respectively. Nutritional profile as crude starch, fiber, protein, ash, and fat ranged from 56.3%–50.80%, 16.50%–11.73%, 16.20%–11.53%, 2.8%–2.1%, and 2.63%–1.63%, respectively. The mineral composition in terms of Mg (527–616 mg/kg) was higher in the landlines followed by Ca (312–368 mg/kg), Na (122.6–146.6 mg/kg), Fe (43.3–65.6 mg/kg), and Zn (22.5–26.6 mg/kg). It was concluded that the indigenous barley landlines had immense nutritional potential and functional attributes. Thus, it can be used for value‐added food products and the development of cottage industry in the region.

## Graphical abstract

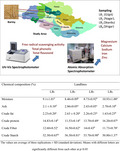

## 1 INTRODUCTION

Barley belongs to the Poaceae family, grown and consumed in Africa, Asia, semi‐arid tropics, and also grown in Europe, America, and Australia (Erkan et al., 2006). The crop possesses health‐promoting nutritional and functional properties (Cook, 2013; Idehen et al., 2017). The main components of barley are carbohydrates with low fat, protein, minerals, vitamins especially vitamin E, dietary fiber, and antioxidants predominantly polyphenols (Das et al., 2016). The nutritional constituents of barley consist of health‐promoting starch (65%–68%), protein (10%–17%), free lipids (2%–3%), β‐glucans (4%–9%), and minerals content ranges from 1.5%–2.5%, respectively. Moreover, total dietary fiber varied from 11%–34% among which, 3%–20% is soluble dietary fiber (Guo et al., 2020; Izydorczyk et al., 2000). The cereal crop also contains nonstarch polysaccharides which are β‐glucan, arabinoxylans, and cellulose, which change the energy content of barley (Das et al., 2016).

Different types of phytochemicals including phenolic acids, flavonoids, lignans, vitamin E, sterols, and folates have been reported in barley. These phytochemicals have health‐promoting attributes such as improvement in reproduction, proper growth, and development of the human body, and also protect the consumer from foreign pathogens, parasites, and predators (Dykes & Rooney, 2007; Lattanzio et al., 2006; Malik, 2012). Also, the cereal crop contains low lipid content with predominant fatty acids as palmitic, oleic, linoleic, and linoleic acid while a higher amount of linolenic acid is present in barely, as compared to wheat. Similarly, the cereal also contains a significant amount of fat‐soluble vitamin E and vitamin B complex (Pitzer, 2009). Some major elements like phosphorus, potassium, calcium, magnesium, sulfur, selenium, and sodium have also been detected in the grains (Das et al., 2016).

Nearly 65% of barley throughout the world is employed for animal feed formulations, 33% for malting application while only 2% is processed as a human diet (Sullivan et al., 2013). The main reason for less production is improper crop safety and its application as fodder (Naheed et al., 2015). However, the crop shows great adaptability and tolerance against the unfavorable environment; therefore, it is successfully grown even on high altitudes of the Himalayas and in the Arctic Circle region (Zhu, 2017). Worldwide annual production was recorded 144 million tons in 2014, and the countries regarding top production are Russia, France, Germany, Australia, and Ukraine (Giraldo et al., 2019). Nevertheless, like food, barley is common in those areas where the other cereals cannot be produced and used in breakfast, making bread, Asian noodles, bars, muffins, biscuits, and cookies and as soap thickener (Izydorczyk & Dexter, 2008; Kremer & Ben‐Hammouda, 2009).

Barley was the staple food of Gilgit‐Baltistan (GB) along with buckwheat, millets, and sorghum till the early sixties. However, with the advent of early wheat varieties, its cultivation and uses gradually declined. Therefore, the present study aimed to determine nutritional and phytochemical composition among the local barley landlines grown at four different districts of Gilgit‐Baltistan. The present study will provide baseline data for utilization of barley and development of by‐product for the local economy and nutritional security.

## 2 MATERIAL AND METHODS

### 2.1 Samples collection and preparation

The current study was conducted in the Advanced Instrumental Laboratory of Karakorum International University. The samples of dried barley LB_1_ (landline barley) Gilgit, LB_2_ (Nagar), LB_3_ (Skardu), and LB_4_ (Shigar) were collected from the farmers in different districts of Gilgit‐Baltistan, Pakistan. The collected samples were cleaned manually for foreign residues and other impurities. After that, the samples were grounded in flour (Mesh size) with the grinding mill, and the final product (weight) was stored in polythene bags for further analysis under ambient conditions.

### 2.2 Free radical scavenging activity

The total antioxidant characteristics of all the samples were detected by following DPPH (2, 2‐diphenyl‐l picryl hydrazyl) technique reported by (Mareček et al., 2017) with minor changes. In detail, DPPH solution was prepared and then followed by covering with aluminum foil and stored under refrigeration temperature for its further use. For antioxidant estimations, 5 g of each barley flour sample was homogenized and then extracted by using methanol (10 ml) for 48 hr. Further, in a volumetric flask (100 ml volume), 0.1 ml extract and 3.9 ml of DPPH solution having 6 × 10^–5^ mol/L concentration was mixed and then incubated at ambient temperature for 35 min. After the incubation time, the absorbance was calculated at 517 nm with UV spectrophotometer. The antioxidant attributes were estimated by employing the expression as under:

$$\text{Inhibition (}\%)=\frac{\text{Blank absorbance}‐\text{sample absorbance}}{\text{Blank absorbance}}\times 100.$$

### 2.3 Total phenolic content

Total phenolic values were recorded by following the Folin–Ciocalteu procedure, as described by Shahzad et al. (2020) with minor modifications. Briefly, 5 g of the barley sample was first ground into powder followed by homogenization and then extracted by using 10 ml methanol for 48 hr. Further, 1 ml (mg/ml) extract was mixed gently with 4.6 ml distilled water and 1 ml Folin–Ciocalteu (1N). After 3 min, 3 ml sodium carbonate (2%) was mixed into the mixture and stand it for 2 hr. Finally, the absorbance was recorded at 760 nm by using a UV spectrophotometer.

### 2.4 Total flavonoid content

The total flavonoid values of the barley samples were tested by according the method described by Manzoor et al. (2019) with minor modification. 1 ml of the samples extract (1 mg/ml) was taken and mixed with 4ml distilled water. Further, 0.3 ml AlCl_3_ (10%) and 2ml of the NaOH (1N) were also poured into the reaction flask. Again, 2.7 ml of distilled water was added, agitated well, and then, absorbance was recorded at 510 nm. Various concentrations of quercetin were employed as an internal standard.

### 2.5 Nutritional composition

#### 2.5.1 Moisture

The moisture content of barley flour was examined by using the protocol set by AACC (2000) Method No. 44–15.02. In detail, 5 g flour was first placed in a petri dish and put in an oven having 105 ± 5°C temperature under vacuum, till a constant weight of the dried sample was achieved. The moisture results were recorded by using the formula as under:

$$\text{Moisture (}\%)=\frac{\text{Weight of original flour sample - Weight of dried flour sample}}{\text{Weight of original flour sample}}\times 100.$$

#### 2.5.2 Crude starch

The crude starch was measured according to the procedure given by Ahmed et al. (2014) by weighing 2 g of barley flour and boiling it with calcium chloride solution in a fuiopopen beaker, stirring it continuously with adding water to maintain the liquid level. After 30 min, it was cooled to room temperature and added 10 ml of stannic chloride solution. Then, filter it through Whatman filter paper, and angular rotation was measured using a 100 mm polarimeter tube, and starch was calculated according to the following formula:

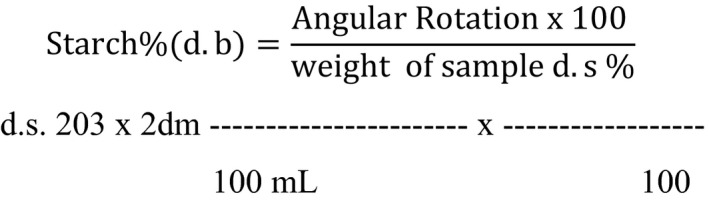

#### 2.5.3 Crude fiber

The crude fiber for samples was analyzed by following the protocol proposed by AACC (2000) Method No. 32–10.01. Briefly, 3 g fat‐free barley flour was first digested with H_2_SO_4_ (1.25%) followed by washing with distilled water, and filtration was performed. Another digestion was done with 1.25% NaOH solution washed with distilled water followed by filtration. Further, ignition of the sample residue was performed. It was done by keeping the digested samples in a muffle furnace at 550–650°C for 3–5 hr. Finally, gray or white ash was acquired for crude fiber analysis. The crude fiber was estimated by employing the expression as under:

$$\text{Crude Fiber (}\%)=\frac{\text{Weight of residue left}‐\text{Weight of ash}}{\text{Weight of sample}}\times 100.$$

#### 2.5.4 Crude protein

For crude protein analysis, the Kjeldahl method was followed, set by AACC (2000) Method No. 406–10.01. In detail, 5g sample was first placed in digestion tube along with 20 ml H_2_SO_4_ (98% pure) and 2 digestion tablets as a catalyst. The digestion was continued for 3–4 hr till the sample appears transparent. Then, the digested samples' temperature was reduced to room temperature and 50 ml volume makeup was done by dilution with water. Moreover, the ammonia trapped in H_2_SO_4_ was removed by addition of NaOH (40%) solution using distillation and collected in a flask having boric acid (4%) solution, methyl red indicator, and titrated against standard N_2_SO_4_ (0.1 N) solution. The crude protein results were recorded by the multiplication of nitrogen (%) with a conversion factor of 5.57.

$$\text{Nitrogen}\;\left(\%\right)=\frac{\text{The volume of}\;0.1\;N\;H_{2}{\mathrm{SO}}_{4}\times \text{Volume of}\;\text{dilution}}{\text{The volume of distillate taken}\times \text{Weight of sample}}\times 100.$$

Crude Protein(%)=Nitrogen(%)×6.25.

#### 2.5.5 Crude fat

For crude fat determination, dried samples were processed in the soxhlet method. In which, continuous refluxing was done by using petroleum ether as solvent as reported by AACC (2000) Method No. 30–10.01. In detail, a 3 g sample was weighed and dried in an oven till constant weight. The dried sample was then wrapped in filter paper and put in soxhlet apparatus and 5 to 6‐time washings were given with petroleum ether as extraction solvent. The solvent was evaporated after extraction, and fat content was determined by employing the formula mentioned below.

$$\text{Crude Fat (}\%)=\frac{\text{Weight of fat in the sample}}{\text{weight of sample}}\times 100.$$

#### 2.5.6 Crude ash

For Ash content analysis, AOAC (2006) Method No.923.03 was followed. Firstly, ignited the empty crucibles at 550°C, weighed and, then, cooled in a desiccator to room temperature.

Then, took a 2 g homogeneous sample in a crucible and placed it in a muffle furnace at 660°C until light gray mass was achieved. Finally, the crucibles were removed from the furnace and allowed to cool down in a desiccator. Calculate the weight of ash along with the crucible and calculate the net weight. Ash content was recorded by using the formula as under;

$$\text{Ash (}\%)=\frac{W3‐W1}{W2}\times 100.$$

whereas; *W*_1_ = crucible weight; *W*_2_ = sample weight; *W*_3_ = sample weight after ashing.

### 2.6 Mineral contents

The mineral content of the barley flour was ascertained by employing the wet digestion method proposed by AOAC (2006). For which, 0.5 g premixed sample was first digested at 60–70°C, by using HNO_3_ (10 ml) in a conical flask for 20 min on a hot plate. Then, redigestion was performed at 190°C by employing 5 ml HClO_4_ (60%) until the flask appeared transparent. Further, the digested samples were poured into the volumetric flasks (100 ml volume), and then, the volume was adjusted with double distilled water followed by filtration The filtered solution was investigated by using atomic absorption spectrophotometer (AA 240 Varian, Australia). Standards of known concentrations were first to run for each mineral, and a standard curve was plotted. The mineral contents of the samples were calculated by employing the respective standard curve prepared for each element. All samples were tested for sodium, potassium, calcium, and iron content with a flame photometer and atomic absorption spectrophotometer (Sherwood Flame Photometer 410), as described by AOAC (2006).

### 2.7 Statistical analysis

All measurements were carried out in triplicates, and it was analyzed with the help of statistics 8.1 (Tallahassee FL 32,317, USA). One‐way analysis of variance (ANOVA) was applied in factorial design at *p* <.05 choose as significant.

## 3 RESULTS AND DISCUSSION

### 3.1 Antioxidant activity of landline barley samples

The DPPH radical scavenging activity of landline barleys from different districts is presented in Table 1. The findings depict that the antioxidant activity of LB_2_ was significantly higher as compared to other landline barley samples (*p* <.05). The DPPH radical scavenging ability was observed highest in LB_2_ (60.3%) followed by LB_3_ (56.3%), LB_1_ (55.6%), and LB_4_ (50.3%). However, no significant difference between LB_1_ and LB_3_ (*p* <.05). Our results were in line with the findings of (Shen et al., 2018). In that study, highland barley variety Zangqing 2000 had 67.53% of bound DPPH radical scavenging ability, higher than other Xinhua and Shangri‐la varieties. Variation in antioxidant activity and concentration of polyphenols in barley vary according to varieties, growth location, environmental factors, and years of growth (Abdel‐Aal et al., 2012; Lahouar et al., 2014; Narwal et al., 2016).

**TABLE 1:** Phytochemical composition of different barely landlines

Phytochemical content	Landlines			
	LB_1_	LB_2_	LB_3_	LB_4_
Free radical scavenging activity (%)	55.6 ± 3.74^b^	60.3 ± 2.58^a^	56.3 ± 3.55^ab^	50.3 ± 1.52^c^
Total phenolic content (mg/g)	1.9 ± 0.70^c^	3.1 ± 0.16^a^	2.9 ± 0.95^b^	1.2 ± 0.10^d^
Total flavonoid content (mg/g)	0.41 ± 0.02^c^	0.55 ± 0.01^a^	0.48 ± 0.02^b^	0.47 ± 0.02^b^

Note

The values are average of three replications ± *SD* (standard deviation). Means with different letters are significantly different from each other at *p* < .05.

### 3.2 Total phenolic and flavonoids components of barley samples

The total phenolic contents of landline barley samples from different districts are presented in Table 1. The findings for the tested parameters from different districts were significantly different from each other (*p* <.05). The total flavonoid content was estimated highest in LB_2_ (3.1 mg/g) followed by LB_3_ (2.9 mg/g), LB_1_ (1.9 mg/g), and LB_4_ (1.2 mg/g). The findings of the current study were quite similar to the study conducted by Abidi et al.(2015), which reported 47–123 mg CE/100 g. Similarly, our findings were slightly higher than those of Bellucci et al. (2013), calculated as 26.9 mg/100g in Dutch barley. The quantity and quality of polyphenols may be affected by some factors such as plant genetics and cultivar, soil type, growing methods, maturity stage, and postharvest management (Taranto et al., 2017). Flavonoid content in barley changes according to variety; white, blue, and purple kernels have a high concentration of flavonoid among others (Liu et al., 2013).

The results about total flavonoid contents (TFC) among different landlines are depicted in Table 1. The obtained results revealed that the investigated parameter in LB_2_ was significantly higher than other barley samples from other districts. The highest total flavonoid contents were determined in LB_2_ as 0.55 mg/g while the lowest total flavonoid contents were recorded in LB_1_ (0.42 mg/g). However, no significant difference between LB_3_ and LB_4_ was observed. Our outcomes were in agreement with Lahouar et al. (2014), they reported results ranging from 195.02 to 220.11 mg gallic acid equivalent/100g fresh weight.

Moreover, the results obtained by Yang et al. (2018) in various highland barley varieties varied from 336.29–453.94 mg/100g, slightly higher than our results, whereas slightly lower outcomes (70–195 mg GAE/ 100 g) than our results were recorded by Abidi (2015), the difference in these results might be due to the variation in varieties, cultivation methods, environmental conditions, and also depends on solvents used during extraction (Abdel‐Aal et al., 2012).

### 3.3 3 Nutritional composition

The chemical composition of landline barley samples in different districts is depicted in **Table** **2**. Findings for the moisture content were significantly higher in the LB_4_ (10.93%) than in other samples (*p <*.05). However, no significant difference between LB_1_, LB_2,_ and LB_3_ was recorded. Our moisture results are in line with the results (7.34%–16.82%) reported by Tavakoli et al. (2010), in barley grains while Bader Ul Ain et al. (2018) calculated the parameter in barely from 10.2%–11.4%. These differences might be due to a variety of differences, storage conditions, geological change, and water holding capacity.

**TABLE 2:** Chemical composition of different barley landlines

Chemical composition (%)	Landlines			
	LB_1_	LB_2_	LB_3_	LB_4_
Moisture	9.1 ± 1.01^b^	8.46 ± 0.89^b^	8.73 ± 0.92^b^	10.93 ± 1.00^a^
Ash	2.1 ± 0.10^d^	2.86 ± 0.05^a^	2.43 ± 0.05^c^	2.70 ± 0.10^b^
Crude fat	2.23 ± 0.20^a^	2.63 ± 0.20^a^	2.26 ± 0.25^a^	1.63 ± 0.25^b^
Crude protein	14.83 ± 0.14^b^	11.53 ± 0.14^d^	13.70 ± 0.49^c^	16.20 ± 0.07^a^
Crude Fiber	12.60 ± 0.52^c^	16.50 ± 0.62^a^	14 ± 0.43^b^	11.73 ± 0.70^c^
Crude Starch	52.30 ± 0.65^bc^	56.30 ± 0.81^a^	53.70 ± 0.98^b^	50.80 ± 1.37^c^

Note

The values are average of three replications ± *SD* (standard deviation). Means with different letters are significantly different from each other at *p* < .05.

Moreover, the results regarding ash content showed significant differences (*p <.05*) among the tested samples. The highest ash content was analyzed in LB_2_ (2.86%) followed by LB_4_ (2.70%), LB_3_ (2.43%), and LB_1_ (2.1%), whereas the minimum value was recorded in LB_1_ to be 2.1%. These findings were closely related to the results of Brennan and Cleary (2005). They assessed total ash content in whole grain barley ranging 1.5%–2.5%. Furthermore, our findings were also in line with a study conducted by Quinde‐Axtell and Baik (2006). They determined ash content as 2%–3% in the barley samples.

The crude fat content for LB_4_ was significantly lower than other samples. However, there was no significant difference between LB_1_, LB_2_, and LB_3_ samples were recorded. Our findings were closely related to Brennan and Cleary (2005), who reported 2%–3% total lipids in barley, whereas Quinde‐Axtell and Baik (2006) detected slightly lower results than our findings.

Also, the crude protein test showed a significant difference among all samples. The highest crude protein was analyzed in the LB_4_ (16.20%) followed by LB_1_ (14.83), LB_3_ (13.70), and LB_2_ (11.53) (*p* <.05). Brennan and Cleary (2005) studies revealed that the protein content in the barley samples fluctuated from 10%–17%. In another study, Suriano et al. (2018) found that the total protein content was 12.75%, similar to our results. The crude fiber content in the LB_2_ (16.50%) was significantly higher than all other barley samples (*p* <.05), whereas no significant difference between LB_1_ and LB_4_ samples was also detected. Also, Quinde‐Axtell and Baik (2006) determined total dietary fiber in barley varieties varied from 11%–34%, the results were in agreement with our study.

The results regarding crude starch showed a significant difference among landlines Barley samples collected from different districts (*p* < .05). The crude starch content in the LB_2_ delivered maximum value (56.3%) followed by LB_3_ (53.7%), LB_1_ (52.3%), and LB_4_ (50.8%). These changes might be due to genetic differences and cultivar (Wozniak et al., 2014). The nutritional composition of cereal grains might be affected by the environmental conditions under they grow and many studies have shown differences in concentration of fat, protein, and β‐glucan content in oat and barley grown under different environment (Redaelli et al., 2013). Moreover, Ping et al. (2013) also reported that the total starch content among 112 Chinese varieties varied from 45.7% to 66.4%. Similarly, Asare et al. (2011) found that the starch content in 10 Canadian barley genotypes ranged from 58.1% to 72.2%. The difference in compositional attributes might be due to environmental conditions like rainfall, temperature, soil type, fertility, and genetic factors (Quinde‐Axtell & Baik, 2006; Rodehutscord et al., 2016). According to Rodehutscord et al. (2016), the chemical composition and physical features of cereals vary with fluctuation in environmental conditions like rainfall, temperature, soil type, fertility, and genetic factors. Quinde‐Axtell and Baik (2006) have also similar views that nutritional parameters in barley may change according to environmental conditions and other factors.

### 3.4 Mineral composition

The mineral composition among different barley landlines is presented in Table 3. The results presented that Na content in the LB_2_ (146.6 mg/kg) and LB_4_ (142.6 mg/kg) was significantly higher than those of LB_2_ and LB_4_ (*p* < .05), whereas the Ca content in the LB_1_ (368 mg/kg) was significantly higher than in other samples (*p* < .05). The Mg content in the LB_2_ (618.6 mg/kg) was significantly higher than all other samples collected from other districts (*p* < .05). The Fe content was notably higher in LB_1_ (65.6 mg/kg) as compared to other landline samples collected from different districts (*p* < .05). The Zn content in the LB_4_ (26.5 mg/kg) was significantly higher than those of LB_2_ and LB_3_ (*p* < .05), whereas there was no significant difference among LB_1_, LB_2_, and LB_3_.

**TABLE 3:** Mineral composition of different barely landlines

Mineral Composition (mg/kg)	Landlines			
	LB_1_	LB_2_	LB_3_	LB_4_
Sodium (Na)	122.6 ± 3.05^c^	146.6 ± 2.08^a^	136.3 ± 4.04^b^	142.6 ± 2.51^a^
Calcium (Ca)	368 ± 1.73^a^	312 ± 1.00^d^	327 ± 2.00^c^	359.6 ± 2.08^b^
Magnesium (Mg)	599.6 ± 3.05^c^	618.6 ± 3.05^a^	609.3 ± 3.05^b^	527 ± 3.00^d^
Iron (Fe)	65.6 ± 1.52^a^	53.6 ± 1.15^b^	51.3 ± 2.51^b^	43.3 ± 2.88^c^
Zinc (Zn)	25.1 ± 0.84^ab^	22.9 ± 1.65^b^	22.5 ± 2.29^b^	26.5 ± 1.50^a^

Note

The values are average of three replications ± *SD* (standard deviation). Means with different letters are significantly different from each other at *p* <.05.

Additionally, Na content in barley samples varied from 56–285 mg/kg was reported by Yan et al. (2016), and the results are in line with our study findings. Whereas slight lower results for different mineral elements (as compared to our results) was determined by Šterna et al. (2015), estimating Na fluctuated from 18.1–20.8 mg/kg, Mg content ranging from 1,123.7 to 1,210 mg/kg, and Ca values differed from 309.33 to 353 mg/kg.

Similarly, Yan et al. (2016) found that the Fe content in different barley samples ranged from 39.5–235.5 mg/kg, whereas Ma et al. (2004) revealed that the Fe content varied 40–60 mg/kg among different varieties of barley. The variations in the mineral composition may be due to environmental changes, landlines, or other factors. Furthermore, MALEKI et al. (2011) demonstrated that the mineral composition of barley grain can be varied according to environmental conditions and fertilizing system. Similarly, Rodehutscord et al. (2016) stated that environmental conditions like rainfall, temperature, soil fertility, and genetic makeup influence the nutritional composition and physical features of cereals.

## 4 CONCLUSION

The current study demonstrated that the local barley landline is a good source of nutrition as well as functional properties with good antioxidant activity. Thus, it is suggested that the crop should be an essential part of our diet and also used for making value‐added products. The current study further provides baseline data for future research in the food and pharmaceutical aspects of barley landlines.

## CONFLICT OF INTEREST

The authors declare no conflict of interest.
